# Intelligent biology and medicine in 2015: advancing interdisciplinary education, collaboration, and data science

**DOI:** 10.1186/s12864-016-2893-x

**Published:** 2016-08-22

**Authors:** Kun Huang, Yunlong Liu, Yufei Huang, Lang Li, Lee Cooper, Jianhua Ruan, Zhongming Zhao

**Affiliations:** 1 Department of Biomedical Informatics, The Ohio State University, Columbus, OH 43210 USA; 2 Department of Medical and Molecular Genetics, Indiana University School of Medicine, Indianapolis, IN 46202 USA; 3 Center for Computational Biology and Bioinformatics, Indiana University School of Medicine, Indianapolis, IN 46202 USA; 4 Department of Electrical and Computer Engineering, The University of Texas at San Antonio, San Antonio, TX 78249 USA; 5 Department of Biomedical Informatics, Emory University, Atlanta, GA 30322 USA; 6 Department of Biomedical Engineering, Emory University / Georgia Institute of Technology, Atlanta, GA 30322 USA; 7 Department of Computer Science, The University of Texas at San Antonio, San Antonio, TX 78249 USA; 8 Center for Precision Health, School of Biomedical Informatics, The University of Texas Health Science Center at Houston, Houston, TX 77030 USA

## Abstract

We summarize the 2015 International Conference on Intelligent Biology and Medicine (ICIBM 2015) and the editorial report of the supplement to BMC Genomics. The supplement includes 20 research articles selected from the manuscripts submitted to ICIBM 2015. The conference was held on November 13–15, 2015 at Indianapolis, Indiana, USA. It included eight scientific sessions, three tutorials, four keynote presentations, three highlight talks, and a poster session that covered current research in bioinformatics, systems biology, computational biology, biotechnologies, and computational medicine.

## Introduction

The 2015 International Conference on Intelligent Biology and Medicine (ICIBM 2015) brought together more than one hundred twenty researchers and trainees from multiple countries with highly interdisciplinary background such as biology, medicine, computer science, biomedical engineering, statistics, mathematics, and chemistry, among others, participated this exciting three-day event. The event provided a forum for attendees to exchange new ideas, demonstrate new tools, showcase recent innovative work, and foster interdisciplinary research collaborations. It was also an important educational and training venue for students and junior investigators in bioinformatics, systems biology, intelligent computing, and computational medicine.

The scientific program included eight scientific sessions covering seven important areas, four tutorials, four keynote presentations, nine highlight talks, and a poster session. Among the scientific sessions, since there are many interesting and important work related to the area of next generation sequencing data analysis, it was split into two sessions held on different days. The detailed information of all presentations and posters can be found on the conference website [1] and in the conference program book. Here we briefly review the keynote speakers’ presentation, followed by the tutorials, and the papers selected by BMC Genomics in the regular scientific sessions.

### Keynote lectures

Four keynote speakers who are world-renowned leaders in bioinformatics, medical informatics, integrative genomics, systems biology and computational medicine delivered lectures on their cutting-edge research, provided insightful views for their research fields, and shared their perspectives on the future of these fields in the era of big data. These speakers were Dr. Jiajie Zhang from The University of Texas Health Science Center at Houston, Dr. Christopher Sanders from Harvard Medical School, Dr. Keith Dunker from Indiana University, and Dr. Sylvia Plevritis from Stanford University. Below, we briefly introduce the four keynote presentations.

“*Beyond Big Data: New Trends in Biomedical Informatics Research and Education*” by Dr. Jiajie Zhang. Dr. Zhang is the Dean, the Glassell Family Foundation Distinguished Chair in Informatics Excellence, and the Dr. Doris L. Ross Professor at The University of Texas School of Biomedical Informatics. He is an elected fellow of the American College of Medical Informatics. Dr. Zhang in his presentation provided insights on the rapidly emerging new trends in biomedical informatics beyond the current Big Data Revolution. He pointed out that Big Data itself cannot solve any problems, instead what the community needs are precise, small scale, transparent, live knowledge and intelligence for the right problem of a right individual at the right time and the right location, with the power of prediction and intervention. In addition, Dr. Zhang discussed these new trends in biomedical informatics research and education as the promise of data in medicine and biology will not be realized without a new generation of students, researchers, and developers who are trained with the state-of-the-art tools and equipped with the most up-to-date knowledge in biomedical informatics.

“*Systems Biology in Action: Prediction of Large Protein 3D Structures and Design of Cancer Combination Therapy*” by Dr. Christopher Sander. Dr. Sander was Head of the Computational Biology Center at Memorial Sloan Kettering Cancer Center and Tri-Institutional Professor at Rockefeller and Cornell Universities. Dr. Sander is a pioneer and world leader in computational biology. In this presentation, he presented his recent high impact work on systems pharmacology, computational genomics, and protein structure and function. First, he introduced perturbation biology, a method for computing responses to combinatorial therapy aiming to block the emergence of resistance to targeted cancer therapies. In addition, he presented cBioPortal for cancer genomics in collaboration with Niki Schultz and Ethan Cerami being information resource architects under his leadership. Finally, he showed recent work on solving the 3D protein folding prediction problem using statistical physics and information from next-generation sequencing in collaboration with Debora Marks at Harvard Medical School and the Zecchina group at the Politecnico di Torino.

“*Intrinsically Disordered Protein and the Origins of Complex Multicellular Organisms*” by Dr. Keith Dunker. Dr. Dunker is a renowned protein scientist and a professor of Biochemistry and Molecular Biology and was the Director of the Center for Computational Biology and Bioinformatics at Indiana University. In this presentation, Dr. Dunker focused on understanding two important biological questions for evolution of multicellular organisms: First, which molecular functions underpinned the evolution of multicellular organisms? Secondly, which of these molecular functions depend on the intrinsically disordered proteins (IDP)? Answers to the first question involve the advent of molecules for cellular adhesion, cell-cell communication, developmental programs, spatial and temporal regulation of the developmental programs as well as cell-specific biochemistry. For the second question, Dr. Dunker and his colleagues used key-words in Swiss Protein ranked for associations with predictions of protein structure or disorder and found that “differentiation” was the biological process most strongly associated with IDPs. In addition, all of the aforementioned five underpinning molecular functions for multicellularity were found to strongly depend on IDP-based mechanisms. These findings lead to new direction in characterizing the evolution of complex multicellular organisms and using gene regulatory network models currently used to explain cellular differentiation.

“*Optimizing Combination Cancer Therapy Based on Single Cell Analysis*” Presented by Dr. Sylvia Plevritis. Dr. Plevritis is a Professor of Radiology in the School of Medicine and (by courtesy) of Management Science and Engineering in the School of Engineering at Stanford University. She is co-Section Chief of Integrative Biomedical Imaging Informatics (IBIIS) at Stanford University, Director of the Stanford Center for Cancer Systems Biology (CCSB) and Director of the Cancer Systems Biology Scholars (CSBS) postdoctoral training program.

Tumors often evolve under exposure to different treatment regimes, leading to intratumor heterogeneity and different clinical outcomes. Personalized treatment strategies are thus required to improve the effectiveness of cancer drug combination therapy. In this study, Dr. Plevritis and her colleagues aim to identify optimal cancer drug combinations based on characterization of an individual patient’s intratumoral heterogeneity in response to a screening panel of single drugs at the single cell level. Specifically, Dr. Plevritis presented mass cytometry, the state-of-the-art single cell technology, to elucidate the intratumoral response to drug exposure. This approach is based on a set of novel algorithms to analyze the high- dimensional data generated by the mass cytometry. The ultimate goal of this study is to optimize cancer drug combinations for each patient by capturing an individual patient’s intratumoral heterogeneity through integrative single-cell based analyses based on proteomic, genomic and imaging data.

### Tutorials

ICIBM 2015 included three tutorial sessions covering frontier and emerging research topics such as next-generation sequencing data analysis, proteomics, and bioimage informatics. These tutorials were well attended and appreciated by the conference participants. They provided a wealth of information on these cutting-edge areas and techniques in bioinformatics and computational biology.

*“Advanced Data Mining and Quality Control of High Throughput Sequencing Data”* (provided by Dr. Yan Guo from Vanderbilt University): Illumina high throughput sequencing (HTS) technology is one of the most prevalent high throughput sequencing technologies driving genomic studies. However HTS data creates numerous bioinformatics challenges due to its complexity, yet simultaneously offer exciting new opportunities for mining new biomedical informatics from the vast amount of data. In this tutorial, Dr. Guo discussed two major aspects of HTS data analysis: quality control (QC) and data mining. QC is critical for downstream analysis but a common myth is that quality control is only needed at the raw data. In fact, QC for HTS data needs to be carried out in at least three stages: raw data, alignment, and variant calling. In this tutorial, Dr. Guo introduced the concept of multi-perspective QC and discussed the detailed QC strategies for both RNA and DNA sequencing data. HTS data with proper QC also provide unique opportunities for data mining, and the specific type of data mining depends on the specific sequencing methods. Dr. Guo specifically introduced potential minable components from both exome and RNA-seq data and discussed the appropriate approach for each of the components.

*“Novel genotype-phenotype associations in human cancers enabled by advanced molecular platforms and computational analysis of whole slide images”* This tutorial was organized by Dr. Lee Cooper from Emory University and Dr. Kun Huang from the Ohio State University. Advances in computing, imaging, and genomics have created new opportunities for exploring relationships between histology, molecular events, and clinical outcomes using quantitative methods. Slide scanning devices are now capable of rapidly producing massive digital image archives that capture histological details in high resolution. Commensurate advances in computing and image analysis algorithms enable mining of archives to extract descriptions of histology, ranging from basic human annotations to automatic and precisely quantitative morphometric characterization of hundreds of millions of cells. These imaging capabilities represent a new dimension in tissue-based studies, and when combined with genomic and clinical endpoints, can be used to explore biologic characteristics of the tumor microenvironment and to discover new morphologic biomarkers of genetic alterations and patient outcomes. In this tutorial, Drs. Cooper and Huang described recent developments in quantitative pathology imaging and illustrate how image features can be integrated with clinical and genomic data to investigate fundamental problems in cancer. Using motivating examples from the study of gliomas (GBMs), Dr. Cooper also demonstrated how public data from The Cancer Genome Atlas (TCGA) can serve as an open platform to conduct *in silico* tissue-based studies that integrate existing data resources.

*“Computational Challenges in Top-Down Proteomics”* This tutorial is offered by Dr. Xiaowen Chen from School of Informatics and Computing at IUPUI. While the genome provides the blueprint of gene products, proteins are the bricks and mortar of biology. Mass spectrometry (MS) is the core technology for the studies of proteins and post-translational modifications. Over the past decade, proteomics has been dominated by bottom-up MS that digests proteins into fragments and analyzes the resulting short peptides. Since information about intact proteins is lost during digestion, recent studies advocated top-down MS that analyzes intact proteins and gives rise to many computational challenges. While top-down MS researchers have made great progress, the algorithms for interpreting top-down MS data are still in their infancy. We describe computational challenges and combinatorial algorithms for the analysis of top-down MS data and show how they enable new biological applications.

### Scientific sessions and BMC Genomics / Systems Biology supplement issues

ICIBM 2015 had eight regular scientific sessions covering recent research in the areas of bioinformatics, systems biology, intelligent computing, and computational medicine. The detailed information of the sessions, including session chairs, authors, presenters, and the title and abstract of each talk were made available on the conference website [1] and in the conference program brochure. The presentations were selected through a rigorous review process from more than 60 submissions based on their scientific merit and technical quality by a program committee of more than 90 experts in the field (see the Conference Organization section below). These sessions were:

- Session I: NGS Data Analysis, I
- Session II: Systems Biology
- Session III: NGS Data Analysis, II
- Session IV: Integrative Genomics
- Session V: Genomics and Genetics
- Session VI: Epigenomics, Proteomics and Metabolomics
- Session VII: Biomarker Discovery and Precision Medicine
- Session VIII: Pharmacogenomics and Systems Medicine

Here we present the editorial report for the supplement to BMC Genomics that includes 20 research papers. Each selected manuscript was reviewed for two rounds. The first round of reviewed was carried out by at least three reviewers and was substantially revised according to reviewers’ critiques. The revision was further reviewed by at least two reviewers before being accepted into the supplement issue. These papers cover a wide spectrum of topics in bioinformatics and computational biology. Below we group the papers in this special issue of BMC Genomics into five categories and summarize them.

### Next and the third generation sequencing data analysis methods

While next generation sequencing (NGS) techniques have been widely adopted by biomedical community during the past decade, new experimental techniques and even the third-generation sequencing technology keep proposing new challenges for data analysis. During the conference, several groups proposed new methods or methods comparison for processing and analyzing NGS and third generation sequencing data. In [2], Feng et al. proposed a Bayesian inference-based method that takes advantage of the signal distributions of electrical voltages measured for all the homopolymers for third generation sequencing technology such as the Nanopore sequencer. By cross-referencing the length of homopolymers in the reference genome and the voltage signal distributions, the proposed integrated model significantly improves the alignment accuracy around the homopolymer regions. Cherukuri and Janga [3] then benchmarked available assembler algorithms such as de Bruijn graphs, Overlap Layout Consensus (OLC) and Greedy extension approaches to find an appropriate framework that can efficiently assemble Nanopore sequenced reads. Their analysis unveiled that OLC-based algorithms could generate a high quality assembly with ten times higher N50 & mean contig values as well as one-fifth the number of total number of contigs compared to other tools. The findings should help in stimulating the development of novel assemblers for handling Nanopore sequence data. In [4], the study aimed to evaluate the appropriateness of different statistical distributions on modeling sequence-context-dependent DNA sequencing error rates generated by different NGS technologies. Using a generalized linear model framework, Hao et al. found that zero-inflated negative binomial distribution fits the sequencing errors the best and also performed the best in identifying low-frequency single nucleotide variants (SNVs), especially within the 0.5 % to 1 % ranges in two commonly used sequencing platforms with completely different biochemistries - Ion Proton and Illumina MiSeq. This work provides guidance in predicting sequencing errors and facilitates low-frequency SNV detection as well as their downstream applications. The development of single-cell RNA sequencing enables tracking cell heterogeneity and determination of cell subpopulations. Chen et al. [5] developed a gene expression variation model (GEVM), utilizing the relation between coefficient of variation (CV) and average expression level to address the over-dispersion of single-cell data, and its corresponding statistical significance to quantify the variably expressed genes (VEGs). Obtaining VEGs allowed researchers to detect possible subpopulations, providing further evidences of cell heterogeneity. In Cui et al. [6], the authors developed a novel algorithm for uncovering the potential types of m6A methylation by clustering the degree of m6A methylation peaks in MeRIP-Seq data. This algorithm utilizes a hierarchical graphical model to model the reads account variance and the underlying clusters of the methylation peaks. It was applied to two different MeRIP-seq datasets and revealed a novel pattern that methylation peaks with less peak enrichment tend to clustered in the 5’ end of both in both mRNAs and lncRNAs, whereas those with higher peak enrichment are more likely to be distributed in CDS and towards the 3’end of mRNAs and lncRNAs. These results suggest that m6A’s functions could be location specific.

### Applications of sequence and NGS data analysis methods

In addition to developing novel methods for analyzing data generated from new NGS techniques or the third-generation technology, application of NGS technologies to biological and medical problems also calls for extensive bioinformatics research. Bai et al. [7] has investigated the variation of gene expression in blood transcriptome profile of Chinese Holstein cows associated to the milk yield traits. Totally, 100 differentially expressed genes (DEGs) between 13 high yielders and 10 low yielders were obtained, which were shown to be significantly enriched in immune response processes. Furthermore, alternative splicing analysis demonstrated that the alternative 3’ splicing site was the major splicing pattern in high yielders, however, in low yielders was exon skipping. This study allowed us to explore associations between immune traits and production traits related to milk production. In [8], Zhou et al. identified 197 exons whose BMSC splicing patterns were altered by LPS via comparing RNA-seq data from LPS-treated samples versus the control. Functional analysis of these alternatively spliced genes demonstrated significant enrichment of phosphoproteins, zinc finger proteins, and proteins undergoing acetylation. Additional bioinformatics analysis strongly suggest that LPS-induced alternatively spliced exons could have major effects on protein functions by disrupting key protein functional domains, protein-protein interactions, and post-translational modifications. The study provides greater understanding of the intracellular mechanisms that underlie the therapeutic potential of BMSCs. The evolution of exceptionally powerful transporter systems in Streptomyces has enabled their adaptation to the complex soil environment. A better understanding of transport systems will allow enhanced optimization of production processes for both pharmaceutical and industrial applications of Streptomyces. In [9], Zhou et al. presented a catalog of transport systems in eleven Streptomyces species and found that each of the species possesses a rich repertoire of transport proteins, which can be divided into a wide range of transporter families. To characterize the biological and medical significance of Bacillus sp. NRRL B-14911, in particular, cardiac autoimmunity, Massilamany et al. [10] sought to analyze the complete genome sequence of this bacterium. The genome was found to encode several virulence factors like adhesins, invasins, colonization factors, siderophores and transporters. The availability of complete genome sequence of this bacterium may facilitate genetic manipulations to assess gene functions associated with bacterial survival and virulence, and also to establish a disease model to study the immune pathogenesis of bacterial myocarditis. In [11], Bai et al. presented an improved algorithm “Read-Split-Run” (RSR) for detecting genome-wide Ire1α-targeted genes with non-canonical spliced regions at a faster speed. They compared the RSR algorithm to the “Read-Split-Walk” (RSW) algorithm when applied to mouse embryonic fibroblast cells (MEF) and the human Encyclopedia of DNA Elements (ENCODE) RNA-seq data. The new RSR algorithm outperformed others in the defined context and showed a higher efficiency in identifying novel splice junctions genome-wide.

### Integrative genomics and precision medicine

One important issue in precision medicine is how to effectively integrate multiple modalities of biomedical data, especially different sets of high throughput molecular data to better stratify patients into groups with distinctive clinical outcomes such as different prognosis and response to treatment. During this conference, a series of papers proposed different integrative genomic methods to achieve this goal. While whole exome-sequencing is widely used to screen for somatic mutations in cancer patients, the mutations often do not occur on the same genes among patients. In [12], Zhang et al. developed a novel approach of integrating patient somatic mutation, transcriptome and clinical data to mine underlying functional gene groups that can be used to stratify cancer patients into groups with different clinical outcomes. Specifically, distance correlation metric was used to mine the correlations between expression profiles of mutated genes from different patients. With this method, a stable subgroup of breast cancer patients that are highly enriched with ER-negative and triple-negative subtypes were identified, and the somatic mutation genes they harbor were capable of acting as potential biomarkers to predict patient survival in several different breast cancer datasets, especially in ER-negative cohorts which has lacked of reliable biomarkers. The method provides a novel and promising approach of integrating genotype and gene expression data in patient stratification in complex diseases. Proper cell models for breast cancer primary tumors have long been the focal point in the cancer’s research. In [13], a comprehensive comparison in copy number variation (CNV), mutation, mRNA expression and protein expression between 68 breast cancer cell lines and 1375 primary breast tumors is conducted and presented. The important drug targets, ESR1, PGR, HER2, EGFR and AR have a high similarity in mRNA and protein in both tumors and cell lines. A total score developed from the four correlations among four molecular profiles suggests that cell lines, BT483, T47D and MDAMB453 have the highest similarity with tumors. In [14], Wang et al. proposed an integrative genomics approach to explore the functional consequences of a key driver gene, *PBRM1* through its truncated mutations in clear cell renal cell carcinoma (ccRCC) by incorporating somatic mutations, mRNA expression, DNA methylation, and microRNA expression profiles from The Cancer Genome Atlas (TCGA). Their results suggested that methylation and microRNA alterations were likely the downstream events associated with the *PBRM1* truncation mutations. This study provided some important insights into the understanding of tumorigenesis driven by *PBRM1* truncated mutations in ccRCC.

### Cancer biomarker discovery and pan cancer study

Discovery of biomarkers and signatures is an important issue in translational research for cancers. During this conference, several studies focused on the methods for identifying cancer specific or common signatures and markers predicting clinical outcomes for cancer patients. For instance, to classify cancer classes (e.g. subtypes) using patient gene expression profiles when both systematic and condition-specific biases presented, Ma et al. [15] developed a novel algorithm called CrossLink (CL). CL exploits the fact that the signature is unique to its associated class under any condition and thus employs an unsupervised clustering algorithm to discover this unique signature. The results showed that CL can achieve robust and improved performance than state-of-the-art normalization algorithms. In [16], the authors performed a pan-cancer analysis of copy number of variants (CNVs) and gene expression in one of the most important gene categories, tumor suppressor genes (TSGs), in order to provide a systematic view of CNV and gene expression concordant changes in TSGs across all the major cancers. They found that 81 TSGs with concordant copy number loss events and decreased gene expression in the tumor samples and provided a draft landscape of CNV in pan-cancer. In [17], Zhang and Chen presented a peptidomics method for identifying cancer-related and isoform-specific peptide for clinical proteomics application from LC-MS/MS. They showed that the method for identifying cancer-specific protein isoform biomarkers from clinical proteomics application is an effective one for increasing the number of identified alternative splicing isoform markers in clinical proteomics.

### Translational bioinformatics and pharmacogenomics

Translational bioinformatics methods including network analysis are widely applied to human disease studies and pharmacogenomics applications. In [18], Chen et al. developed a drug repositioning approach combining human disease genomics and mouse phenotype data towards predicting targeted therapies for glioblastoma (GBM). For existing GBM drugs, this approach achieved a significantly higher median rank than a recent approach (9.2 % vs. 45.6 %). In addition, many top predictions have been demonstrated effective in inhibiting the growth of human GBM cells. In [19], Li et al. extracted the functional modules and identified 19 key rifampin-response genes that are associated with seven function pathways that include drug response and metabolism, and cancer pathways. In addition, six genes functioning as gene hubs in the gene networks that are regulated by rifampin was identified. The results suggest that rifampin contributes to changes in the expression of genes by regulating key molecules in the protein interaction networks. In [20], Xu and Wang presented an integrated approach for drug repurposing for rheumatoid arthritis (RA). They developed a network-based ranking algorithm to find diseases that shared high degrees of genetic commonality with RA and then implemented a drug prioritization algorithm to reposition drugs from RA-related diseases to treat RA. This approach performed significantly better in novel predictions than the existing approach when evaluated using 165 not-yet-FDA-approved RA drugs. While gene co-expression network analysis is widely adopted, there is a lack of a rigorous way to evaluate the concordance of the expression profiles for the genes in co-expressed modules. Han et al. [21] presented a linear algebraic based Centralized Concordance Index (CCI) for evaluating the concordance of co-expressed gene modules from gene co-expression network analysis. The CCI can be used to evaluate the performance for co-expression network analysis algorithms as well as for detecting condition specific co-expression modules.

### Conference organization

#### 2015 International conference on intelligent biology and medicine (ICIBM 2015)

(November 13–15, 2015, Indianapolis, Indiana, USA)

Our sincerest thanks to the members of our Steering, Program, Publication, Workshop/Tutorial, Award, Publicity, and Local Organization committees, as well as our numerous reviewers and volunteers, for their countless hours and energy spent on making ICIBM 2015 a success! The conference would not make so many accomplishments if the support and efforts from those people were not provided.

Sponsors Indiana University, Center for Computational Biology and Bioinformatics at Indiana University School of Medicine, Vanderbilt University, Bioinformatics Resource Center at Vanderbilt-Ingram Cancer Center, The University of Texas at San Antonio, Shanghai Center for Bioinformation Technology, China.

General Chairs Zhongming Zhao (Vanderbilt University and now The University of Texas Health Science Center at Houston) and Yunlong Liu (Indiana University).

Steering Committee Lang Li (Indiana University), Yufei Huang (The University of Texas at San Antonio), Mathew Palakal (Indiana University), Tony Hu (Drexel University), Han Liang (MD Anderson Cancer Center), Subha Madhavan (Georgetown University).

Program Committee Chair: Kun Huang (The Ohio State University), Co-Chair: Lee Cooper (Emory University), Members: Kristen Anton (Dartmouth College), Yong-sheng Bai (Indiana State University), William S. Bush (Vanderbilt University), Jake Chen (Indiana University -Purdue University Indianapolis), Xue-Wen Chen (Wayne State University), Yidong Chen (University of Texas Health Science Center at San Antonio), Jianlin Cheng (University of Missouri Columbia), Juan Cui (University of Georgia), Qinghua Cui (Peking University, China), Youping Deng (Rush University Medical Center), Joshua Denny (Vanderbilt University), Jeremy Edwards (University of New Mexico Health Sciences Center), Weixing Feng (Harbin Engineering University), Jennifer M. Fettweis (Virginia Commonwealth University), Marcelo Fiszman (National Library of Medicine, National Institutes of Health), Jan Freudenberg (Feinstein Medical Research Institute), Ge Gao (Peking University, China), Chittibabu (Babu) Guda (University of Nebraska Medical Center), Yan Guo (Vanderbilt University), Hao Han (Singapore Bioinformatics Insistute), Zhi Han (The Ohio State University),, Tzu-Hung Hsiao (University of Texas Health Science Center at San Antonio), Weichun Huang, (National Institute of Environmental Health Sciences), Yang Huang (Kaiser Permanente), Yufei Huang (The University of Texas at San Antonio), Jenn-Kang Hwang (National Chiao Tung University, Taiwan), Peilin Jia (The University of Texas Health Science Center at Houston), Yufang Jin (The University of Texas at San Antonio), Victor Jin (The University of Texas Health Science Center at San Antonio), Sun Kim (Seoul National University, Korea), Dmitry Korkin (University of Missouri - Columbia), K.B. Kulasekera (University of Louisville), Jun Kong (Emory University), Fuhai Li (Houston Methodist hospital Research Institute), Lang Li (Indiana Univeristy), Leping Li (National Institute of Environmental Health Sciences), Li Liao (University of Delaware), Honghuang Lin (Boston University), Chunyu Liu (University of Chicago), Hongfang Liu (Georgetown University), Qi Liu (Vanderbilt University), Tianming Liu (University of Georgia), Yin Liu (The University of Texas Health Science Center at Houston), Yunlong Liu (Indiana University), Zhandong Liu (Baylor College of Medicine), Xinghua Lu (University of Pittsburgh), Zhiyong Lu (NCBI, National Library of Medicine), Patricio A. Manque (Universidad Mayor, Chile), Tabrez Mohammad (The University of Texas Health Science Center at San Antonio), Hatice Gulcin Ozer (The Ohio State University), Ranadip Pal (Texas Tech University), Yonghong Peng (University of Bradford, United Kindom), Horacio Perez-Sanchez (Catholic University of Murcia, Spain), Jiang Qian (Johns Hopkins University), Thomas Rindflesch (National Institutes of Health), Jianhua Ruan (The University of Texas at San Antonio), Bairong Shen (Soochow University, China), Alexander Statnikov (New York University Langone Medical Center), Jingchun Sun (The University of Texas Health Science Center at Houston), Wing-Kin Sung (National University of Singapore, Singapore), Manabu Torii (Georgetown University Medical Center), Vladimir Uversky (University of South Florida), Jun Wan (Johns Hopkins University), Edwin Wang (National Council of Canada/McGill University), Jing Wang (Vanderbilt University), Junbai Wang (Oslo University Hospital, Norway), Qingguo Wang (Sloan-Kettering Memorial Institute), Yufeng Wang (The University of Texas at San Antonio), Xiaoyan Wang (University of Connecticut), Chaochun Wei (Shanghai Jiao Tong University), Xiwei Wu (City of Hope National Medical Center), Yonghui Wu (The University of Texas Health Science Center at Houston), Junfeng Xia (Anhui University, China), Lu Xie (Shanghai Center for Bioinformation Technology, China), Hua Xu (The University of Texas Health Science Center at Houston), Jianhua Xuan (Virginia Tech), Bin Xue (University of South Florida), Zhenqing Ye (The University of Texas Health Science Center at San Antonio), Sungroh Yoon (Seoul National University, Korea), Bing Zhang (Vanderbilt University), Jie Zhang (The Ohio State University), Michelle Zhang (The University of Texas at San Antonio), Yanqing Zhang (Georgia State University), Min Zhao (University of the Sunshine Coast, Australia), Yanjun Zhao (Troy University), Zhongming Zhao (Vanderbilt University), Huiru (Jane) Zheng (University of Ulster, United Kingdom), Wenjin Jim Zheng (The University of Texas Health Science Center at Houston), and Dongxiao Zhu (Wayne State University).

Publication Committee Chair: Jianhua Ruan (The University of Texas at San Antonio), Co-Chair: Sarath Janga (Indiana University), Milan Radovich (Indiana University).

Workshop/Tutorial Committee Chair: Ting Wang (Washington University), Co-Chair: Xiaowen Liu (Indiana University).

Award Committee Chair: Hua Xu (The University of Texas Health Science Center at Houston), Co-Chair: Yufei Huang (The University of Texas at San Antonio).

Publicity Committee Chair: Dongxiao Zhu (Wayne State University).

Local Organization Committee Chair: Yunlong Liu (Indiana University).
